# Arrhythmic burden of long-term postsurgical hypoparathyroidism assessed with 24-hour Holter ECG

**DOI:** 10.1007/s12020-025-04235-6

**Published:** 2025-05-09

**Authors:** Rachele Santori, Vito Menafra, Alessandro Sgreccia, Luciano Colangelo, Cristiana Cipriani, Marco Occhiuto, Giovambattista Desideri, Evaristo Ettorre, Salvatore Minisola, Jessica Pepe

**Affiliations:** Department of clinical, internal, anesthesiology and cardiovascular sciences, “Sapienza” University of Rome, Rome, Italy

**Keywords:** Arrhythmia, Hypoparathyroidism, 24-hour Holter ECG, Heart rate variability

## Abstract

**Purpose:**

Postsurgical hypoparathyroidism is associated with an increased cardiovascular risk, however an increased arrhythmic risk in this population is controversial.

**Methods:**

Twenty-two postmenopausal women with postsurgical hypoparathyroidism and 22 healthy postmenopausal women of the same age were enrolled. Each subject underwent blood tests, standard 12-lead ECG, 24-hour Holter ECG and echocardiographic measurements. The exclusion criteria were: previous cardiovascular disease, arrhythmias, diabetes mellitus, kidney failure, use of drugs that may interfere with cardiac conduction.

**Results:**

Time since diagnosis of postsurgical hypoparathyroidism was 19.33 ± 8.82 years. As expected, serum calcium and PTH levels were significantly lower in hypoparathyroid patients compared to controls, while phosphorus was higher (all *p* < 0.05). ECG parameters were within normal values in both groups. A higher number of hypoparathyroid patients had significantly more supraventricular and ventricular premature beats compared to controls (*p* < 0.05). An index of heart variability, predominantly of parasympathetic activity, such as the root mean square of the difference between successive RR intervals was significantly lower in hypoparathyroid patients compared to controls (21.09 ± 9.84 vs 33.0 ± 17.89 msec, *p* = 0.009). All the echocardiographic parameters were within the normal limits. The only statistically significant difference between groups was a lower ejection fraction (EF) in the hypoparathyroid group compared to controls (57.5% ± 2.98 vs 64.85% ± 6.09, *p* < 0.0001). A longer time since diagnosis of hypoparathyroidism was only positively associated with heart rate: B = 0.45, QRS: B = 0.66 and negatively with EF:B = −0.64 (all *p* < 0.05).

**Conclusion:**

24-hour ECG Holter demonstrated an increase in arrhythmia in postmenopausal women with long-term postsurgical hypoparathyroidism in the absence of long QT or cardiac structural abnormalities.

## Introduction

Postsurgical hypoparathyroidism is one of the most frequent causes of hypocalcemia and low serum levels of parathyroid hormone [1, 2]. It is a disease caused by an unintentional removal or injury to parathyroid tissue at the time of an anterior neck operation.

A recent systematic review of the literature reported numerous complications of this disease in different organs, in particular at the cardiovascular level [3]. An Italian national epidemiological study showed that cardiovascular events were the third complication most associated with hospitalizations of both non-surgical and postsurgical hypoparathyroid patients [4].

The increased cardiovascular risk is linked to several physiopathological mechanisms that are not yet well understood. Hypocalcemia induces QT interval prolongation which predisposes to ventricular arrhythmias [5]. Furthermore, serum values outside the recommended calcium range (8–9 mg/dL), both hyperphosphatemia (>4.5 mg/dL) and especially an increase in the calcium-phosphorus product (>55 mg^2^/dL^2^) [6] can be associated with a higher frequency of peripheral vascular calcifications [7], coronary calcification [8], increase in carotid IMT [9] and valvular calcification in this population.

However, there are some population-based studies that found no increased risk of cardiovascular disease in patients with postsurgical hypoparathyroidism compared to controls [10, 11].

This discrepancy may be due to inclusion of both male and female subjects with different cardiovascular risk factors, or to different length of the disease, or to different therapy with calcitriol and calcium supplements, or to different cardiovascular outcomes included, as shown by some, but not all studies [4, 12].

In fact, a recent meta-analysis showed an increase in cardiovascular risk, but not an increased arrhythmic risk in this population [13].

It would be of interest to know the prevalence of arrhythmias during the activities of daily life in this population. A recent study showed an increase in premature ventricular beats, evaluated with 24-hour Holter ECG, in a small sample of premenopausal females and males with hypoparathyroidism [14]. There are no studies in postmenopausal women with long-term postsurgical hypoparathyroidism.

The aim of the study is the arrhythmic assessment with 24-hour Holter ECG in postmenopausal women with long-term postsurgical hypoparathyroidism compared to healthy controls of same age and sex.

## Methods

Twenty-two postmenopausal women with postsurgical hypoparathyroidism, attending the Bone Unit of Sapienza University of Rome for osteoporosis screening from 2014–2024, were enrolled.

The exclusion criteria were: severe valvular disease, history of left ventricular hypertrophy, history of significant arrhythmias, left bundle branch block, cardiac pacemaker implantation, ventricular pre-excitation, diabetes mellitus, glomerular filtration rate <60 mL/min, use of drugs that may interfere with cardiac conduction and mineral metabolism, hypothyroidism/hyperthyroidism not pharmacologically corrected (except for calcium and calcitriol needed to treat hypoparathyroidism) and obesity (BMI > 30 Kg/m^2^). Patients were not taking, for the treatment of hypertension, beta blockers or calcium antagonists, nor spironolactone that may interfere with QT conduction.

Each subject underwent blood tests, standard 12-lead ECG, 24-hour Holter ECG and echocardiographic measurements. Healthy women of the same age and sex, who had participated in a previous study (*n* = 26) that had performed the same tests, were considered as controls, if they matched for both age and BMI (*n* = 22) [15].

In each patient, on the day of the 24-hour Holter ECG recording, a fasting blood sample was taken in the morning to measure serum calcium levels, phosphate, creatinine, vitamin D, parathyroid hormone and TSH as previously described [16].

The 12-lead resting electrocardiogram was performed using a device with dedicated software (PC ECG Norav 1200 Medical Ltd), as previously described [17]. The corrected QT interval (QTc) was calculated using the Bazett formula (QTc = QT/√RR).

A 24-hour Holter electrocardiogram was performed using a DR300 Holter device from Northeast Monitoring Inc. as previously described [15].

A study of the cardiac autonomic nervous system was also performed by analyzing the variability of the heart rate (HRV) in the time domain during the 24-hour Holter ECG recording. The parameters studied were: SDNN (standard deviation of the RR intervals), an index of both orthosympathetic and parasympathetic activity, pNN50 (percentage of RR intervals of consecutive sinus beats with a variation >50 msec) and rMSSD (root mean square of the difference between successive RR intervals), both indexes of predominantly parasympathetic activity [18].

Transthoracic echocardiogram was performed using Mindray ultrasound imaging device, dc-80, x-insight, within 10 days since 24-hour Holter ECG recording, to measure the parameters as previously described [19].

Left ventricular end-diastolic diameter (LVEDD), interventricular septal thickness (IVS), posterior wall thickness (PWT) and left atrial (LA) transverse diameter were measured according to the recommendations of the American Society of Echocardiography [20].

Ejection fraction (EF%) was calculated according to Simpson’s rule. Early diastolic transmitral flow velocities (E) and during atrial contraction (A) and their ratio (E/A) were also measured. Left ventricular mass (LVM) and left ventricular mass indexed to body surface area (LVMI) were estimated based on the linear dimensions of the left ventricular cavity and wall thickness at end diastole.

The study was approved by our local Ethics Committee and was performed in accordance with the ethical standards as laid down in the 1964 Declaration of Helsinki and its later amendments.

### Statistical analysis

The statistical program SPSS version 22.0 for Windows was used for data analysis. The results of the descriptive statistics were expressed as mean ± standard deviation (SD). Between-group differences at baseline were assessed by Student’s t-test. Frequencies were compared using chi-squared test. Pearson correlation coefficient was used to assess the relationship between continuous variables. The results were considered significant for values of *p* < 0.05. Because there are no studies in postmenopausal hypoparathyroid patients, to determine sample size we relied on the only study assessing 24-hour Holter ECG in hypoparathyroid patients that demonstrated, in premenopausal women and men, a significant increase in arrhythmic risk in a sample of 18 patients. Thus, we decided to include 22 hypoparathyroid patients.

## Results

Anthropometric parameters were not different between patients with hypoparathyroidism and controls. The mean age of hypoparathyroid patients was 62.68 ± 8.07 years and, as expected, serum calcium and PTH levels were significantly lower compared to controls, while phosphorus was higher (all *p* < 0.05, Table 1). The mean calcium phosphorus product was 39.81 ± 6.60 mg^2^/dL^2^, a value that is within the limits of <55 mg^2^/dL^2^ as recommended for the treatment of chronic hypoparathyroidism [21].

**Table 1:** Anthropometric parameters and biochemical values in hypoparathyroid patients and controls

	Hypoparathyroid patients	Controls	*P* values
Age (years)	62.68 ± 8.07	66.5 ± 5.74	0.08
Body mass index (Kg/m^2^)	24.45 ± 5.01	23.96 ± 3.19	0.7
Creatinine (mg/dL)	0.87 ± 0.30	0.81 ± 0.13	0.39
Calcium (mg/dL)	8.77 ± 0.54	9.41 ± 0.36	<0.0001
Phosphorus (mg/dL)	4.78 ± 0.76	3.87 ± 0.49	<0.0001
25(OH) vitamin D (ng/mL)	34.68 ± 17.15	33.39 ± 17.33	0.80
Parathyroid hormone (ng/L)	8.28 ± 4.27	44.70 ± 7.76	<0.0001
TSH (mIU/L)	2.56 ± 1.06	1.90 ± 1.30	0.07

*TSH* thyrotropin

The mean years since diagnosis of postsurgical hypoparathyroidism was 19.33 ± 8.82 years. All patients were taking calcitriol on average 0.64 ± 0.32 mcg daily and calcium carbonate on average 1004.76 ± 578.33 mg daily. Ten patients were taking also cholecalciferol, 800 IU orally per day, in order to achieve a serum vitamin D level of at least 30 ng/mL.

There was no difference between hypoparathyroid patients and controls as concerns cardiovascular risk factors and oral therapy prescribed for these diseases (Table 2).

**Table 2:** Number of patients with cardiovascular risk factors and drugs in hypoparathyroid patients and control group

	Hypoparathyroid patients	Controls	*P* values
Cardiovascular risk factors			
Current smoking	5 (22.7%)	4 (18%)	1
Dyslipidemia	9 (40.9%)	4 (18%)	0.18
Hypertension	10 (45.4%)	7 (31.8%)	0.53
Current medications			
Antihypertensives	7 (31.8%)	4 (18%)	0.48
Antiplatelet	1 (4.5%)	2 (9%)	1
Statins	9 (40.9%)	3 (13.6%)	0.08

The standard ECG parameters were within normal values in both groups and did not differ between the hypoparathyroid patients and controls, with the exception of a shorter p wave compared to controls, but still within the normal range (62.72 ± 19.80 vs 83.72 ± 18.64 msec, *p* = 0.0008, Table 3). Only one hypoparathyroid patient had a basal QTc of 470 msec, which is higher than 460 mms, which is considered the higher cut-off value for a normal QTc in women.

**Table 3:** 12 leads rest ECG parameters in hypoparathyroid patients and in controls

	Hypoparathyroid patients	Controls	*P* values
FC (bpm)	75.86 ± 10.62	70.64 ± 9.3	0.09
P (msec)	62.72 ± 19.80	83.72 ± 18.64	0.0008
PR (msec)	159.54 ± 24.39	157.93 ± 17.94	0.80
QRS (msec)	86 ± 28.35	77.47 ± 11.99	0.20
QTc (msec)	406.63 ± 28.35	409.17 ± 16.76	0.71

Results are presented as mean ± 1 SD. *QTc* QT corrected with Bazett formula, *msec* millisecond

None of the subjects had symptoms or ischemic events during the 24-hour Holter ECG recording. Hypoparathyroid patients had significantly more supraventricular (SVBPs) and ventricular premature beats (VPBs) compared to controls (Table 4). In particular, more than 40% of the patients had SVPBs in triplets compared to none in the control group (*p* < 0.05).

**Table 4:** 24-hour heart rate and number of patients with arrhythmias in hypoparathyroid patients and controls with 24-hour Holter ECG

	Hypoparathyroid patients	Controls	*P* values
Number of patients with SVPBs	22 (100%)	13 (59%)	0.001
Number of patients with VPBs	18 (81.8%)	7 (31.8%)	0.001
24 h heart rate (beats/ min)	78.6 ± 7.9	74.75 ± 6.09	0.07
SVPBs in couplets	14 (63.6%)	7 (31.8%)	0.06
SVPBs in triplets	9 (40.9%)	0	0.001
Runs of more than 4 beats	7 (31.8%)	2(9%)	0.13
SVPBs higher than 76	7 (31.8%)	1(4.5%)	0.04
VPBs in couplets	1 (4.5%)	2(9%)	1
VPBs in triplets	1 (4.5%)	0	1
Runs of more than 4 beats	0	0	1
SDNN (msec)	120.23 ± 41.44	130.62 ± 32.21	0.35
pNN50 (%)	2.88 ± 4.27	3.87 ± 2.36	0.34
rMSSD (msec)	21.09 ± 9.84	33.0 ± 17.89	0.009

*SVBPs* supraventricular and ventricular premature beats, *VPBs* ventricular premature beats, *SDNN* standard deviation of the RR intervals, *pNN50* percentage of RR intervals of consecutive sinus beats with a variation >50 msec and *rMSSD* root mean square of the difference between successive RR intervals, *msec* millisecond

If we consider the absolute number of SVPBs, approximately one third of the patients experienced more than 76 SVPBs compared to one patient in the control group (*p* < 0.05) (Table 4). A significantly higher prevalence of VPBs, 81.8%, were found in hypoparathyroid patients compared to 31.8% of the subjects in the control group (all *p* < 0.05).

As regards the study of heart rate variability, hypoparathyroid patients had a significantly lower rMSSD compared to controls (21.09 ± 9.84 vs 33.0 ± 17.89 msec, *p* = 0.009, Table 4). None of the HRV parameters was associated with biochemical parameters or time since postsurgical diagnosis.

All the echocardiographic parameters were within the normal limits in both groups. The only statistically significant difference between groups was a lower ejection fraction in the hypoparathyroid group compared to controls (57.5% ± 2.98 vs 64.85% ± 6.09, *p* < 0.0001, Table 5).

**Table 5:** Echocardiographic parameters in hypoparathyroid patients and controls

	Hypoparathyroid patients	Controls	*P* values
LVEDD (mm)	48.36 ± 8.56	47.27 ± 14.82	0.76
IVS (mm)	9.15 ± 1.63	9.86 ± 1.55	0.14
PWT (mm)	8.67 ± 1.21	8.98 ± 1.36	0.42
LVMI	80.57 ± 24.05	85.40 ± 16.92	0.44
EF (%)	57.5 ± 2.98	64.85 ± 6.09	<0.0001
E/A	0.94 ± 0.16	0.93 ± 0.21	0.85
LA (mm)	36.77 ± 4.54	36.61 ± 4.12	0.90

Results are presented as mean ±1 SD

*LV* left ventricular, *IVS* interventricular septum thickness, *PWT* posterior wall thickness, E/A velocities of early transmitral diastolic flow (E) and flow velocity during atrial contraction (A), *LA* transverse diameter of left atrium, *LVEDD* left ventricular end-diastolic diameter, *LVMI* left ventricular mass indexed to body surface area

A longer time since diagnosis of hypoparathyroidism was only positively associated with heart rate: B = 0.45, QRS wave: B = 0.66 and negatively with EF: B = −0.64, all *p* < 0.05. Heart rate was positively associate with LVEED: B = 0.50 and SDNN was negatively associated with IVS: B = −0.46, all *p* < 0.05.

## Discussion

Our study showed for the first time an increased arrhythmic risk with 24-hour Holter ECG in postmenopausal women with, on average, 20 years of postsurgical hypoparathyroidism. The strength of the study is the inclusion of asymptomatic hypoparathyroidism patients for arrhythmias with normal echocardiogram and with QTc within normal limits.

Of interest to note, our patients had a normal serum calcium level, well controlled with therapy, although significantly different from controls. It has been shown in a previous study, in hypoparathyroid patients with a ROC analysis, a decrease in the possibility of prolonged QTc at serum total calcium cut-off of 8.3 mg/dL [5]. This probably explains the finding in our population of normal QTc, because the serum mean calcium levels were 8.77 ± 0.54 mg/dL.

During the 24-hour Holter ECG, a higher number of patients had supraventricular and ventricular premature beats compared to controls, in line with the previous findings of higher incidence of premature beats in premenopausal women and men affected by postsurgical hypoparathyroidism [14]. This finding is important because the incidence of ischemic stroke and composite cardiovascular events, from several studies, is associated in the general population with a greater prevalence of premature atrial beats [22]. Moreover, a higher number of hypoparathyroid patients had more than 76 premature beats compared to controls, which has been shown to predict mortality in the general population [23].

The study of heart rate variability showed lower parameters in hypoparathyroid patients, with significant lower rMSSD compared to controls, which is an index of heart variability of predominantly parasympathetic activity.

This finding is in line with a recent study, in hypoparathyroid patients, which evaluated the cardiovascular autonomic system, although through a different method, suggesting that these patients may have an impaired cardiovascular autonomic system compared to controls and hypocalcemia seemed to aggravate its severity [24]. In the literature, a higher risk of mortality has been demonstrated in patients affected by parasympathetic neuropathy, for example in patients with diabetes mellitus [25, 26].

Not only hypocalcemia could be linked to this alteration but also chronic hyperphosphatemia which determines an increase in the phosphaturic factor: fibroblast growth factor 23 (FGF-23). In fact, FGF-23 was independently associated with decreased HRV in patients with chronic kidney disease (stage 5) [27]. However, we did not measure FGF-23 in our study to assess this association, and this could be considered a limit of the study.

Echocardiogram examination showed normal parameters in hypoparathyroid patients. Although ejection fraction was within the normal range, patients with hypoparathyroidism showed a reduced EF compared to controls.

It is interesting to note how the years since diagnosis were significantly associated with a low EF. One hypothesis could be the chronic stimulation of FGF-23 which would favor the damage of cardiovascular structures up to the development of fibrosis and hypertrophy through various mechanisms [28, 29]. In non-hypoparathyroid patients, undergoing elective angioplasty, in multivariable regression analysis, the observed relationship between FGF-23 and left-ventricular function remained significant after adjustment for estimated glomerular filtration rate, presence of left-ventricular hypertrophy, and other confounding variables [30]. Moreover, prevalent atrial fibrillation was associated with elevated FGF-23 levels, while the presence of coronary artery disease was not [30]. However, another recent study did not find association between FGF-23 and carotid plaques, carotid intima media thickness, cardiac valve calcification, and left ventricular hypertrophy in hypoparathyroid patients [31]. In this study, phosphorus was the only significant variable associated with cardiac valve calcification. Thus, the role of FGF-23 in this setting remains controversial.

Considering that our patients had both normal QTc values and normal cardiac structure, we can hypothesize that parasympathetic impairment with low rMMDS may lead to arrhythmia. In addition, the other parameters SDNN (msec) and pNN50 (%) were lower in hypoparathyroid patients compared to controls, although the difference was not statistically significant. High levels of HRV parameters are generally signs of efficient autonomic mechanisms in a healthy individual, while reduced HRV parameters often show an autonomic nervous system malfunction [32]. It has been shown that both sympathetic and vagal control impairment could contribute to ventricular arrhythmogenesis by complex electrophysiological mechanisms in the general population [33]. In a recent study conducted in non hypoparathyroid patients, HRV time-domain parameters were reduced in patients with premature ventricular beats as compared with those without [34].

Consistent with the previous paper that showed an impaired cardiovascular autonomic system [24], we can hypothesize that the finding of lower rMMSD is associated with hypocalcemia, even if we did not find a statistically significant association in our study, probably due to the small number of subjects included.

Our study has some limitations: the inclusion only of female patients of Caucasian origin and no males, the lack of measurement of left atrial volume or atrial and ventricular strain parameters.

In conclusion, our study demonstrates, for the first time, an increase in arrhythmias in postmenopausal women with long-term postsurgical hypoparathyroidism in the absence of long QT or cardiac structural abnormalities. Furthermore, we have shown the lower heart rate variability, in particular for the parasympathetic system with 24-hour Holter ECG.

Further prospective and multicenter studies, with a higher number of patients, including males are needed to try to define the natural history of postsurgical hypoparathyroidism, focusing on the involvement of the cardiovascular system in order to prevent arrhythmias.

## Data Availability

No datasets were generated or analysed during the current study.
